# Smell and Taste Loss Recovery Time in COVID-19 Patients and Disease Severity

**DOI:** 10.3390/jcm10050966

**Published:** 2021-03-02

**Authors:** Athanasia Printza, Mihalis Katotomichelakis, Konstantinos Valsamidis, Symeon Metallidis, Periklis Panagopoulos, Maria Panopoulou, Vasilis Petrakis, Jannis Constantinidis

**Affiliations:** 1 First Otolaryngology Department, Medical School, Faculty of Health Sciences, Aristotle University of Thessaloniki, 54124 Thessaloniki, Greece; kosvals@hotmail.com (K.V.); janconst@otenet.gr (J.C.); 2 Otolaryngology Department, School of Health Sciences, Democritus University of Thrace, Dragana, 387479 Alexandroupoli, Greece; mkatotom@med.duth.gr; 3 First Department of Internal Medicine, AHEPA Hospital, Medical School, Faculty of Health Sciences, Aristotle University of Thessaloniki, 54124 Thessaloniki, Greece; symeonam@auth.gr; 4 Department of Internal Medicine, School of Health Sciences, Democritus University of Thrace, Dragana, 387479 Alexandroupoli, Greece; ppanago@med.duth.gr (P.P.); vasilispetrakis1994@gmail.com (V.P.); 5 Laboratory of Microbiology, School of Health Sciences, Democritus University of Thrace, Dragana, 387479 Alexandroupoli, Greece; mpanopou@med.duth.gr

**Keywords:** olfactory dysfunction, coronavirus disease-2019, COVID-19, loss of smell, anosmia, loss of taste, post-viral, recovery, disease severity, gustatory dysfunction, chemosensory loss

## Abstract

A significant proportion of people infected with SARS-CoV-2 report a new onset of smell or taste loss. The duration of the chemosensory impairment and predictive factors of recovery are still unclear. We aimed to investigate the prevalence, temporal course and recovery predictors in patients who suffered from varying disease severity. Consecutive adult patients diagnosed to be infected with SARS-CoV-2 via reverse-transcription–polymerase chain reaction (RT-PCR) at two coronavirus disease-2019 (COVID-19) Reference Hospitals were contacted to complete a survey reporting chemosensory loss, severity, timing and duration, nasal symptoms, smoking, allergic rhinitis, chronic rhinosinusitis, comorbidities and COVID-19 severity. In a cross-sectional study, we contacted 182 patients and 150 responded. Excluding the critically ill patients, 38% reported gustatory and 41% olfactory impairment (74% severe/anosmia). Most of the patients (88%) recovered their sense of smell by two months (median: 11.5 days; IQR: 13.3). For 23%, the olfactory loss lasted longer than a month. There were no significant differences in the prevalence and duration of chemosensory loss between groups of varying COVID-19 severity, and sexes (all *p* > 0.05). Moderate hyposmia resolved quicker than more severe loss (*p* = 0.04). Smell and taste loss are highly prevalent in COVID-19. Most patients recover fast, but nearly one out of ten have not recovered in two months.

## 1. Introduction

Since the coronavirus disease-2019 (COVID-19) pandemic outbreak, many studies have demonstrated that a significant proportion of people who test positive for COVID-19 have a new onset of smell or taste loss [1,2,3,4]. The Centers for Disease Control and Prevention, the World Health Organization, and National Public Health Authorities added ‘new loss of taste or smell’ to the list of symptoms related to COVID-19. The pathogenesis of anosmia related to SARS-CoV-2 has not been defined and most studies have shown that COVID-19-related olfactory dysfunction demonstrates distinct characteristics differentiating it from post-viral olfactory loss related to other viral causes [1,5]. The olfactory loss is of sudden onset, usually profound, and comes early in the disease process [3,4,6,7]. The duration of the smell and taste disorders in COVID-19 disease is still unclear. Many studies reported a quick recovery in the majority of patients [1,8,9]. However, chronic symptoms after COVID-19 disease, including persisting fatigue and loss of taste and smell, have been reported by patients even several months after the onset of the disease [10,11]. The long-term recovery and the influence of the COVID-19 severity or the chemosensory dysfunction severity on the outcome are not clear. We aimed to investigate the longer-term recovery of smell and taste loss in COVID-19 patients who suffered from varying disease severity and chemosensory impairment severity.

## 2. Materials and Methods

A telephone survey was conducted on consecutive adult patients diagnosed as being infected with SARS-CoV-2 at two COVID-19-Reference University Hospitals, in March and April 2020, in a cross-sectional study. All patients had been diagnosed via a reverse-transcription–polymerase chain reaction (RT–PCR). The study had ethics approval by the two institutional review boards. Three call attempts for each participant were made. All participants provided verbal consent during the interviews. Patients who were not reachable or reported that they did not recall the relevant period events were excluded. Olfactory or/and gustatory disorders before COVID-19 and cognitive disorders were also exclusion criteria. We did not collect data for the deceased patients. The patients were contacted and asked to complete a survey related to taste and smell impairment related to COVID-19 (Table 1. The telephone survey content). It included questions about impairment of smell and taste, nasal congestion, and rhinorrhea. The patients were asked to rate the severity of every symptom on an ordinal scale with the following response options: 0: no loss/absence of the symptom; 1: mild; 2: moderate; 3: severe; 4: extremely severe. The survey also included questions about the timing and duration of symptoms, smoking, history of allergic rhinitis, and chronic rhinosinusitis (CRS). Demographic characteristics (sex and age) and comorbidities were also recorded. Information on severity ratings of COVID-19 was collected from the medical records. The clinical severity of COVID-19 was defined as described by WHO [12] as mild, moderate, severe, and critical. The mild disease includes symptomatic patients meeting the case definition for COVID-19 without evidence of viral pneumonia or hypoxia, moderate patients not exhibiting signs of severe pneumonia, severe patients with clinical or radiographic signs of severe pneumonia including SpO2 < 90% on room air or respiratory rate > 30 breaths/min, and critical ICU-treated patients.

### Statistical Analysis 

Data were analyzed with IBM SPSS Statistics for Windows version 25.0 (IBM Corp., Armonk, NY, USA). Descriptive statistics were obtained; continuous variables are expressed as means with standard deviation, while categorical variables are presented as frequencies (percentages). The normality of the variables was ascertained with the Kolmogorov–Smirnov and Shapiro–Wilk test when the number of data was more or less than 50 respectively. Differences between not normally distributed quantitative data were assessed with the use of the Mann–Whitney U test for independent samples. For differences of qualitative parameters between groups, the Chi-square test was applied. For multiple comparisons between more than two groups of not normally distributed quantitative and qualitative variables, Kruskal–Wallis and Chi-square tests were performed, respectively. No post hoc pairwise comparisons were performed. Correlations between two categorical variables were evaluated either with the use of the Chi-square test or Fisher’s Exact test (in the case of dichotomous categorical variable), and logistic regression was applied to check associations between a categorical and a continuous variable. A *p* value of <0.05 was considered as the statistical significance level.

## 3. Results

We contacted 182 patients. Twenty-six were not reachable, five declined to participate, one had a history of hyposmia. The study cohort consisted of 150 patients (all Caucasian), with a mean age of 51.6 ± 16.8 (ranging from 18 to 89 years). The patients’ demographic and clinical characteristics are presented in Table 2.

The median time from the disease onset to the patients’ survey was 61 days (IQR:13). More than half of the participants had no other medical history (57%) while the most common comorbidities were hypertension, diabetes, and cardiac diseases. The study cohort consisted of patients who had suffered from all disease severity levels.

Olfactory and gustatory disorders were reported by 58 patients (39%) and 54 patients (36%) respectively. Forty-nine patients (33%) reported olfactory and gustatory disorders, nine isolated smell loss and five isolated taste loss. We analyzed further the chemosensory loss prevalence and characteristics in patients with mild, moderate, and severe disease, excluding ICU-treated patients (*n* = 10), since this small subgroup was not considered representative of the critically ill patients for reasons that we comment in the discussion. In this cohort, 41% experienced a loss of smell, which was severe or extremely severe for 74% of them and 38% taste loss (extremely severe for 61% of them) (Table 3. Chemosensory loss characteristics). One out of four patients experienced smell loss before other COVID-19 symptoms. Only a small percentage suffered from nasal blockage and rhinorrhea. 

**Figure 1:**
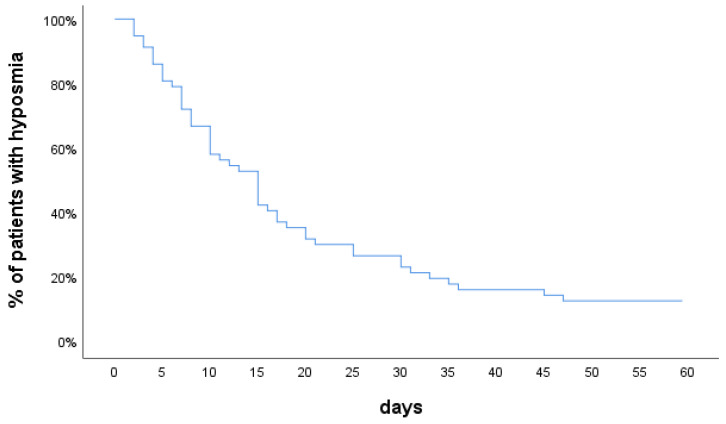
Olfactory recovery over time.

Most of the patients (88%) recovered their sense of smell by 61 days. The median recovery time was 11.5 days (IQR: 13.3), (mean: 14.8 ± 11.2). In two weeks, 58% of the patients had an olfactory recovery and in a month 77%. Similarly, 42 patients (79%) recovered their sense of taste by 61 days. The median recovery time was 10 days (IQR: 8), (mean: 13.8 ± 10.6). Figure 1 shows the recovery time after the onset of smell loss. 

No statistically significant differences were noted in the prevalence of smell loss and taste loss and their duration between groups of varying disease severity (mild, moderate, severe), and sexes (all *p* > 0.05) (Table 4). The percentages of patients who recovered their sense of smell or taste in the subgroups with varying chemosensory loss severity showed no statistically significant differences (all *p* > 0.05) (Table 5). Patients’ groups with varying olfactory loss severity showed statistically significant differences in the days to smell loss recovery (*p* = 0.04). In the patients who recovered their sense of smell, patients with moderate loss had a quicker recovery compared to patients with more severe impairment. The smell loss correlated significantly with the taste loss (Chi-square test, *p* < 0.001), and the presence of rhinorrhea (Chi-square test, *p* = 0.005).

A few participants who suffered olfactory loss reported smoking, allergy, and CRS. Therefore, we did not perform a subgroup analysis regarding the olfactory recovery. Eight patients who developed smell loss were smokers, and 88% of them recovered their sense of smell in an average time of 15.4 days. Six patients with smell loss reported a history of allergic rhinitis. Five out of them (82%) recovered olfaction in 5, 5, 7, 16, and 33 days (an average time of 13.2 days). Only one patient in the subgroup of smell loss reported a history of chronic rhinosinusitis. There were no significant associations between olfactory dysfunction and age (logistic regression, *p* = 0.267), sex (Chi-square test, *p* = 0.12), smoking (Fisher Exact test, *p* = 0.919), disease severity (Chi-square test, *p* = 0.327), allergic rhinitis (Fisher Exact test, *p* = 0.355), chronic rhinosinusitis history (Fisher Exact test, *p* = 0.639) and the presence of nasal blockage (Chi-square test, *p* = 0.059). 

## 4. Discussion

Our cohort exhibited a significant prevalence of smell loss, severe and of sudden onset in most cases. A large proportion of the patients recovered from their chemosensory losses in a month (77%) and even more of them (88%) in two months. A characteristic pattern of quick recovery is evident (six out of ten recovered in two weeks) as it has been reported in other studies [1,13]. However, a small proportion of patients exhibit persisting loss indicating the need to identify predictive factors for persisting hyposmia. No difference was noted in the prevalence of olfactory and gustatory disorders between mild, moderate, and severe COVID-19 disease. Previous studies indicated a greater prevalence of chemosensory deficits in outpatients compared to hospitalized patients [1,14]. It has been postulated that anosmia might be a biomarker of the magnitude of the host’s response to SARS-CoV-2 infection [14]. However, a more detailed analysis of the correlation of smell impairment with disease severity levels is limited to date. We report on a cohort of consecutive patients of all disease severity levels. We did not include the small subgroup of ICU-treated patients in further analysis, because there are serious concerns regarding the validity of self-reporting in this subgroup. The non-ICU-treated patients were representative of home- and hospital-treated patients. Only a small percentage of patients were not reached on the telephone calls, not willing to participate, or presented exclusion criteria. On the contrary, among the critically ill patients, almost half did not survive and a significant proportion of the survivors were excluded from the study because they were still suffering from serious deconditioning. The findings of other research teams are supportive of our results. Recently, a high prevalence of smell impairment (95%) was reported in assisted-breathing patients (ICU-excluded) [15]. Moein et al. also reported no significant relationship between COVID-19 severity and smell impairment in a cohort of hospitalized patients presenting a high prevalence of smell impairment [16]. 

The majority of our patients had severe loss of smell/anosmia, at onset (74%), in agreement with the findings reported in other studies [1,5]. Our patients’ groups with varying chemosensory loss severity showed no statistically significant differences in recovery rates. Vaira et al. also reported no significant difference in the persistence of impairment between patients with varying olfactory loss severity at baseline evaluation of their cohort [7]. However, regarding the time to recovery in those who recovered their sense of smell, in our study, patients with moderate olfactory loss had a quicker recovery compared to patients with more severe impairment. This is in agreement with findings reported by Lechien et al., who found that a less severe loss of smell was significantly associated with an earlier recovery [5]. In our study, the calculated mean duration of smell loss recovery was smaller for extremely severe than for severe loss; however, our anosmic patients did not have a quicker recovery overall. The days to recovery have been calculated and compared only for the patients who recovered their sense of smell by 61 days. Whereas all patients with severe hyposmia (100%) had recovered their sense of smell by 61 days, a percentage of anosmic patients had not recovered. Therefore, the percentage of anosmic patients who had not recovered by 61 days from smell loss onset and had olfactory impairment for longer than 61 days are not included in this comparison. Regarding individual patients results, it took four patients (out of 11) 33, 35, 36, and 45 days to recover olfaction in the severe loss subgroup and four patients (out of 27) 30, 30, 31, and 47 days in the anosmia subgroup. The main difference between these subgroups is the patients that had not recovered by 61 days. Larger studies on patients with all levels of disease severity will be needed to determine whether there are predisposing factors for developing long-lasting chemosensory disorders. 

The prevalence of nasal blockage and rhinorrhea (11.4% and 9.2% respectively) was small, similar to that reported by other studies [17]. There are, though, studies that reported a much higher prevalence of nasal obstruction and rhinorrhea [18]. We found no significant association between olfactory dysfunction and the presence of nasal blockage. Altundag et al., though, reported that nasal congestion was found to be more prevalent in cases with olfactory dysfunction compared to patients without olfactory dysfunction [19]. We found that smell loss significantly correlated with taste loss and rhinorrhea. Other studies have also shown such a correlation [5]. Although the typical COVID-19-related smell impairment usually does not affect patients with significant nasal symptoms, a small percentage of patients might have a component of nasal inflammatory changes contributing to the hyposmia. 

Similar to other studies, we found no associations between age, and gender and smell impairment [7]. The prevalence of allergic rhinitis, chronic rhinosinusitis, and smoking were small in our cohort and no association was found with olfactory loss. A few participants who suffered olfactory loss reported smoking, allergy, and CRS. Therefore, we did not perform a subgroup analysis regarding the olfactory recovery, but descriptive statistics suggest similar patterns of recovery in patients with allergic rhinitis and chronic rhinosinusitis with those recorded in the whole cohort of patients with smell loss. Most studies found no association of comorbidities with the persistence of olfactory dysfunction [5,7], but a recent study reported an association of comorbidities with a worse olfactory recovery in patients with allergic rhinitis, smoking, and hypertension [9].

Most of the patients in our study (88%) had recovered their sense of smell by two months, but a small proportion presents persisting hyposmia. Similar results have been reported by Lechien et al., who reported that, at two months, 80% of their cohort had achieved normal levels of olfactory function [5]. However other researchers have reported higher rates of early recovery (86% in a month after the onset of olfactory dysfunction) [17] or worse recovery [20]. Recently 6-month follow-up data were published on a cohort of patients who presented with a sudden loss of smell in March 2020 reporting persisting very severe and complete loss of smell in 11% of the patients [21]. Fatigue and smell loss were the most common symptoms in a cohort of patients questioned for long-term persistence of symptoms post COVID-19, a mean of 125 days after disease onset [22]. Our knowledge regarding SARS-CoV-2-related symptoms is evolving [23]. Another population-based study found that, in a cohort of non-hospitalized subjects contacted for reporting persistent symptoms, 65% reported a loss of smell and 69% loss of taste at diagnosis and 12% reported loss of smell and 10% loss of taste a median of 117 days from disease diagnosis [24]. 

A strength of our study is the inclusion of a comprehensive cohort of consecutive patients with a confirmed diagnosis of COVID-19 by two reference hospitals, therefore limiting patient selection bias related to age, residence, health-care profession, and information about COVID-19-related smell loss. Our cohort is representative of all disease severity levels. The recovery rate beyond the early four weeks recovery was measured. The chemosensory loss severity was rated at a scale that allowed us to examine the possible correlation of the olfactory and gustatory loss severity with the recovery rate and the time from chemosensory loss onset to recovery. 

A limitation of our study is that the chemosensory dysfunction was not documented with olfactory and gustatory tests. Olfactory questionnaires are considered less reliable in comparison to objective tests. Vaira et al. reported that 10.3% of patients who were found to have a disorder on objective testing had self-reported normal function [7], and adversely in a prospective controlled trial that assessed with validated psychophysical tests the patients’ complaints of smell loss, 61% of COVID-19 patients reported a subjective loss in smell, whereas 54% had a positive test [25]. Self-reporting was appropriate given the retrospective type of our study. The research on COVID-19-related hyposmia relies a lot on questionnaires due to the pandemic restrictions and the short duration of the hyposmia [1]. Another limitation of the study is that patient reports are subjected to recall bias. Furthermore, there is a risk of misclassification of severity ratings when self-reporting of olfactory or gustatory function is retrospective. Recall is considered to be good for distinctive disease symptoms [26]. Smell loss is a very distinct symptom. We acknowledge that rating of symptoms’ severity retrospectively can be inaccurate, but in the context of COVID-19-related smell loss, the great majority of patients in all studies report a sudden and severe change of functional status (severe hyposmia or anosmia) [1] and this reduces the risk of inaccurate rating. Asking the patients to recall events at an order, reference to a calendar, and intervening health events can improve recall [26]. During the pandemic, being diagnosed with COVID-19 was a cardinal health event and with the anxiety of whether the mild disease would turn to more serious, a reference to a calendar of events is strong and a timeline exists for patients regarding the disease resolution. Recall reliability can increase by using precise language, and confirming that the patient is not psychologically or physically impaired [26]. Our study followed these recommendations. We developed a short, appropriate-for-telephone-use survey, using simple everyday language. Furthermore, we excluded from this study patients under rehabilitation for serious deconditioning. In studies about COVID-19-related smell loss, the most appropriate methods of data collection were applied, balancing recruitment bias, recall bias, and the research questions. Another limitation of the study is the small sample size of the subgroups of patients with different COVID-19 severity levels and chemosensory loss. Similarly, the sample sizes of the subgroups of patients with different chemosensory loss severity are small. Differences in the recovery might be detectable in larger participants’ groups.

Smell and taste loss is highly prevalent in COVID-19 of all levels of severity. Most patients recover fast, but one out of ten have not recovered in two months. The recovery rates up to two months do not correlate with the COVID-19 and chemosensory loss severity. The time from chemosensory loss to recovery for the patients who recover is associated with the severity of impairment. Less severe hyposmia tends to resolve quicker.

## Figures and Tables

**Table 1:** The telephone survey content.

During Your COVID-19 Illness	
1. Did you experience loss/impairment of smell?	The severity in an ordinal scale 0–4
2. Did you experience loss/impairment of taste?	The severity in an ordinal scale 0–4
3. Did you experience nasal congestion/obstruction?	The severity in an ordinal scale 0–4
4. Did you experience rhinorrhea?	The severity in an ordinal scale 0–4
5. When did you first notice the loss of smell?	Before/After diagnosis
6. Did the loss/impairment of smell resolve and when?	Yes ● No Days from onset
7. Did the loss/impairment of taste resolve and when?	Yes ● No Days from onset
8. Are you smoking?	Yes ● No ● Ex-smoker ● Electronic
9. Do you have a history of allergic rhinitis?	Yes ● No
10. Do you have a history of chronic rhinosinusitis?	Yes ● No

**Table 2:** The patients’ demographic and clinical characteristics.

	Patients, *n* = 150, *n* (%)
Age, years	18–89
Mean ± SD	51.6 ± 16.8
Sex	
Male	93 (62)
Female	57 (38)
Smoking	21 (14)
Allergic rhinitis	11 (7)
Chronic rhinosinusitis	3 (2)
Comorbidities	
No medical history	86 (57)
Hypertension	30 (20)
Diabetes	16 (11)
Cardiopathy	9 (6)
Covid-19 severity	
Mild	56 (37)
Moderate	50 (33)
Severe	34 (23)
Critical (ICU-treated)	10 (7)
Hospitalized	94 (63)

*n*: number of patients; %: percentage, SD: standard deviation.

**Table 3:** Chemosensory loss characteristics in patients with mild, moderate, and severe COVID-19 disease.

	Patients (*n* = 140), *n* (%)
Smell loss	57 (41)
Taste loss	53 (38)
Smell and taste loss	48 (34)
Nasal obstruction	16 (11)
Rhinorrhea	13 (9)
Allergic rhinitis	11 (8)
Chronic rhinosinusitis	3 (2)
Smell loss severity	
Mild	3 (5)
Moderate	12 (21)
Severe	11 (19)
Extremely severe (anosmia)	31 (54)
Taste loss severity	
Mild	3 (6)
Moderate	11 (21)
Severe	7 (5)
Extremely severe	32 (61)
Smell loss recovery	50 (88)
Taste loss recovery	42 (79)
Hyposmia before other symptoms	15 (26)
Smell loss duration (days), median (IQR)	11.5 (13.3)
Taste loss duration (days), median (IQR)	10 (8)

*n*: number of patients; %: percentage; IQR: Interquartile Range.

**Table 4:** Comparison of the prevalence and duration of hyposmia between groups of varying COVID-19 disease severity and sexes.

	Smell Loss Prevalence Patients; *n* (%)	*p*	Hyposmia Duration Days; Mean ± SD	*p* *
COVID-19 Disease Severity		0.327		0.756 **
Mild Moderate Severe	19 (33) 23 (46) 15 (44)		13.5 ± 8.3 14.1 ± 12 16.9 ± 13.2	
Sex		0.327		0.874 ***
Male Female	33 (38) 24 (44)		14.1 ± 9.2 15.7 ± 13.9	

*n*: number; %: percentage of hyposmic patients in any disease severity or sex subgroup; SD: Standard deviation; *p*: comparison of hyposmia prevalence in the different subgroups, Chi-square test; *p* *: comparison of hyposmia duration in the different subgroups; **: Kruskal–Wallis test; ***: Mann–Whitney U test.

**Table 5:** Comparison of olfactory and gustatory recovery rates and chemosensory loss duration between patients’ groups with varying chemosensory loss severity.

	Recovery Rates Patients; *n* (%)	*p*	Days to Recovery Mean ± SD	*p* *
Smell loss severity		0.396		0.04
Moderate Severe Extremely severe	10 (83) 11 (100) 27 (87)		9 ± 6.8 21.2 ± 12.5 14.7 ± 10.3	
Taste loss severity		0.51		0.084
Moderate Severe Extremely severe	8 (73) 6 (85) 28 (88)		8.5 ± 7 15.8 ± 12.8 14.8 ± 10.7	

*n*: number; %: percentage of patients in any chemosensory severity subgroup who recovered; SD: standard deviation; *p*: comparison of recovery rates between groups of different chemosensory severity, Chi-square test; *p* *: comparison of days to recovery between groups of different chemosensory severity, Kruskal–Wallis test.

## Data Availability

Data are available upon request from the authors.
